# Anti-tumor immunotherapy, radiotherapy, and chemotherapy multimodal combination strategies and interactions in unresectable, locally advanced lung cancer: Clinical practice, mechanism, and biomarker research

**DOI:** 10.1515/jtim-2026-0044

**Published:** 2026-06-13

**Authors:** Yan Xu, Hui Guan, Yuan Wu, Huiyu Huang, Siyuan Yu, Xiaolu Yin, Mengzhao Wang

**Affiliations:** Department of Pulmonary and Critical Care Medicine, Peking Union Medical College Hospital, Chinese Academy of Medical Sciences and Peking Union Medical College, Beijing, China; Department of Radiation Oncology, Peking Union Medical College Hospital; Chinese Academy of Medical Sciences and Peking Union Medical College, Beijing, China; Precision Diagnostics Development, Oncology R & D, AstraZeneca, Shanghai, China

**Keywords:** lung neoplasm, immunotherapy, radiotherapy, biomarker

## Abstract

Immunotherapy has fundamentally transformed the management of lung cancer. For unresectable, locally advanced non-small cell lung cancer (LA-NSCLC) and limited-stage small-cell lung cancer (LS-SCLC), the current standard of care is platinum-based concurrent chemoradiotherapy (cCRT) followed by consolidation immune-checkpoint blockade, a sequence that significantly prolongs progression-free and overall survival. Attempts to deliver immunotherapy concurrently with cCRT have not improved efficacy in randomized trials and have raised additional safety concerns. Induction chemotherapy plus immunotherapy followed by cCRT is biologically rational and feasible, but definitive benefit awaits phase III confirmation. Optimal sequencing, the most effective immunotherapeutic agents, and the best radiotherapy schedules remain active research priorities. The biological rationale for combining radiotherapy and chemotherapy with immunotherapy rests on their capacity to increase tumor immunogenicity, such as by releasing neoantigens, enhancing major histocompatibility complex-I (MHC-I) expression, and promoting dendritic-cell activation, thereby amplifying subsequent T-cell–mediated cytotoxicity. Yet the same cytotoxic stimuli concurrently elicit lymphodepletion, expand regulatory T cells, and trigger pro- inflammatory cytokine cascades that can blunt antitumor immunity. Consequently, the net therapeutic benefit depends on meticulous optimization of sequencing, fractionation, and dosing to harness immunostimulatory efects while minimizing immunosuppressive sequelae. Circulating tumor DNA, metabolic imaging parameters, and real-time immune-profiling are being integrated into non-invasive biomarker platforms that track response and guide post-concurrent chemoradiotherapy (cCRT) immunotherapy decisions. The next generation of curative-intent therapy will be defined by multidisciplinary, patient-centred treatment plans that combine advanced radiotherapy techniques to reduce toxicity, novel agents such as bispecific antibodies or antibody–drug conjugates, biomarker-driven precision medicine, and proactive adverse-event management.

## Introduction

Immunotherapy has changed the treatment landscape of lung cancer.^[1, 2, 3]^ For both non-small cell lung cancer (NSCLC) and small cell lung cancer (SCLC), the use of immune checkpoint inhibitors (ICIs), specifically programmed cell death protein 1 (PD-1)/programmed death-ligand 1 (PD-L1) inhibitors, prolongs the overall survival (OS) of patients. Locally advanced lung cancer is the most challenging and heterogeneous disease,^[4]^ with large differences between patients in treatment and prognosis. With the continuous advancement of ICIs in clinical research, the treatment strategies for patients with locally advanced NSCLC (LA-NSCLC) have undergone significant changes.^[5]^ Perioperative adjuvant/neoadjuvant therapy is a popular topic in clinical research for potentially resectable LA-NSCLC.^[6,7]^ The standard treatment for unresectable LA-NSCLC is concurrent chemoradiotherapy (cCRT) followed by maintenance immunotherapy based on the result of PACIFIC study.^[8, 9, 10, 11]^ Approximately 30% of SCLC is diagnosed at a limited stage, and most of them are locally advanced lung cancer.^[12]^ cCRT followed by immunotherapy is also the current standard treatment for limite stage SCLC (LS-SCLC), which is undergoing transformation due to the emergence of the ADRIATIC model.^[13]^

Despite the recognized potential of enhanced immunotherapeutic efficacy post-cCRT, several scientific questions remain unanswered. For instance, the optimal sequencing and combination of treatments, the role of different immunotherapeutic agents and different radiotherapy regimens, the adverse events of the combined treatment strategy, and the identification of biomarkers that can predict response to therapy are active areas of research. Therefore, the purpose of this review was to evaluate the current literature on anti-tumor immunotherapy, radiotherapy, chemotherapy multimodal combination strategies and interactions in unresectable, locally advanced lung cancer, focusing on clinical applications, mechanisms, and biomarkers.

## Current treatment status of locally advanced lung cancer

### Unresectable locally advanced NSCLC

#### Consolidation immunotherapy after cCRT or sCRT

In the PACIFIC study, unresectable stage IIIA or IIIB NSCLC patients without disease progression after cCRT received durvalumab or placebo as consolidation maintenance therapy.^[8,9]^ Compared to placebo, durvalumab significantly prolonged median progression-free survival (PFS) (16.9 mo *vs*. 5.6 mo; hazard ratio [HR]= 0.55; 95% confidence interval [CI], 0.45 – 0.68;) and median OS (47.5 mo *vs*. 29.1 mo; HR = 0.72; 95% CI, 0.59 – 0.89).^[10]^ This field has received significant research attention (Table 1 and Supplementary Table S1) (Figure 1).

**Figure 1:**
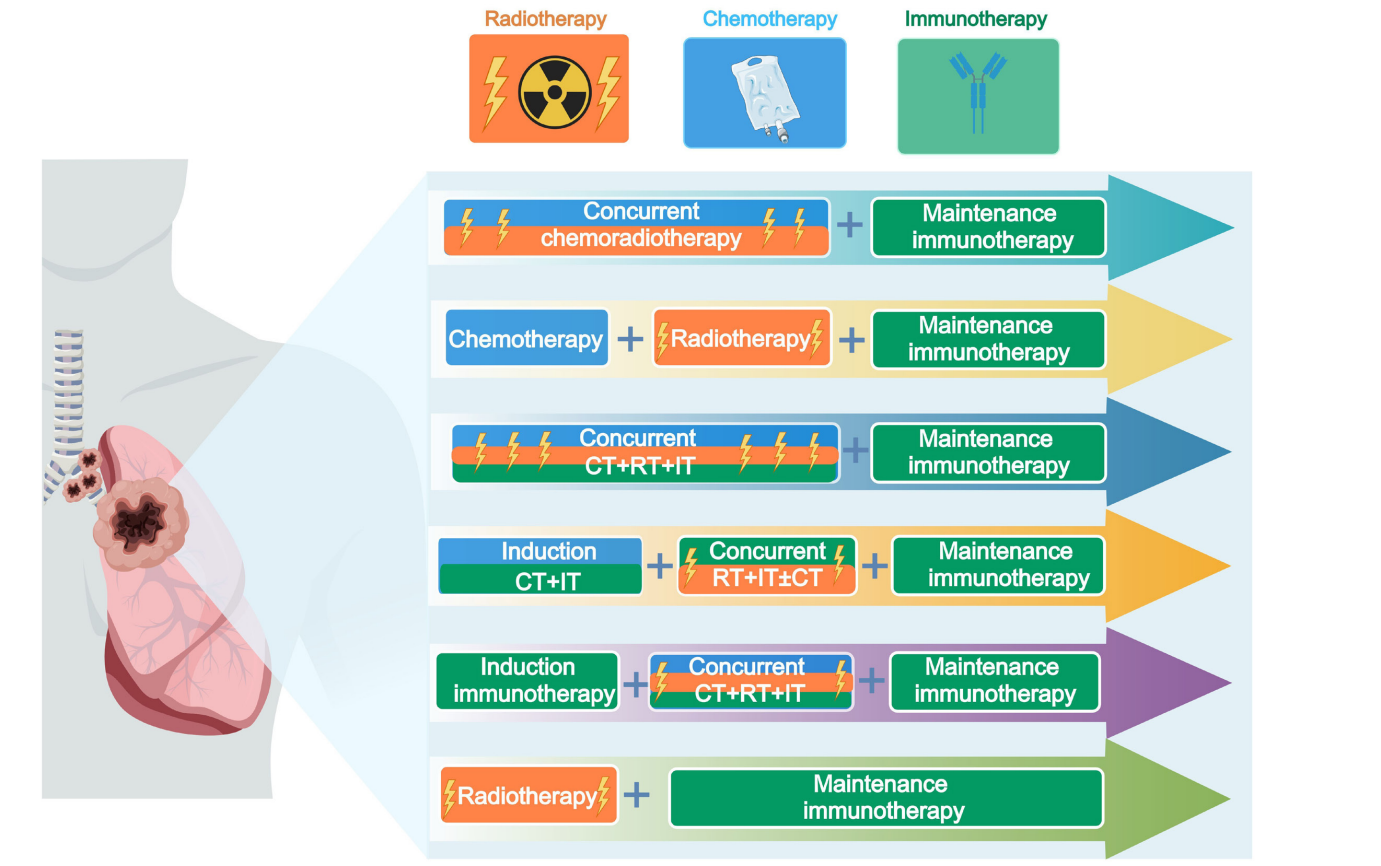
Multimodal combination strategies of anti-tumor immunotherapy, radiotherapy, and chemotherapy in unresectable, locally advanced lung cancer.

**Table 1:** Completed clinical trials of combined immunotherapy, radiotherapy, and chemotherapy strategies for unresectable, locally advanced NSCLC

Trial	Tumor	Phase	Pts	Design	RT	CT	IT	Outcome	AEs
PACIFIC ^[8, 9, 10]^ (NCT021 25461)	Stage III NSCLC	3	713 (2:1)	cCRT→IT *vs*. cCRT	TRT	PBC	Durvalumab *vs*. palcebo	mPFS: 16.9 mo *vs*. 5.6 mo mOS: 47.5 mo *vs*. 29.1 mo	G3-4 RP or Pne 3.4% *vs*. 2.6% Pne 4.4% *vs*. 3.8%
GEMSTONE- 301 (NCT03728556)^[15,16]^	Stage III NSCLC	2	381 (255:126)	cCRT or sCRT→IT *vs*. cCRT or sCRT	TRT	PBC	Sugemalimab *vs*. palcebo	mPFS: 9.0 mo *vs*. 5.8 mo	G3-5 Pne 3% *vs*. < 1 %
PACIFIC-5 (NCT03706690)^[23]^	Stage III NSCLC	3	407 (mITT 252:1 29)	cCRT or sCRT→IT *vs*. cCRT or sCRT	TRT	PBC	Durvalumab *vs*. palcebo	mPFS: 14.0 mo *vs*. 6.5 mo	SAE Pne 5.54% *vs*. 4.48%
LUN 14-179 (NCT02343952)^[14]^	Stage III NSCLC	2	93	cCRT→HT	TRT	PBC	Pembrolizumab	mPFS 1 8.7 mo mOS 35.8 mo	G ≥ 2 Pne 17.2%
SKYSCRAPER-03 (NCT04513925)^[17]^	Stage III NSCLC	3	829 (413:416)	cCRT→IT	TRT	PBC	Tiragolumab + atezolizumab *vs*. durvalumab	mPFS 14.2 mo *vs*. 13.8 mo mOS: 45.6 mo *vs*. 45.8 mo	G3-4 TRAEs 13.8% *vs*. 10.7% G5 TRAEs 0.5% *vs*. 1.7%
R-ALPS (NCT04325763)^[20, 21]^	Stage III NSCLC	3	553 (209, 21 2, 132)	cCRT or sCRT→IT	TRT	PBC	Benmelstovart + anlotinib *vs*. Benmelstobart *vs* placbo	mPFS: B + A 15.1 mo *vs*. P 4.2 mo; B 9.7 mo *vs*. P 4.2 mo; B + A 17.4 mo *vs*. B 11.2 mo	G3-5 RP: B + A 0% *vs*. B 1.9% *vs*. P 2.3% G3-5 infec Pne B + A 8.1 % *vs*. B 4.7% *vs*. P 5.3% G3-5 immune Pne B + A 0% *vs*. B 0.9% *vs*. P 0%
BTCRC-LUN 16-081 (NCT03285321)^[18]^	Stage III NSCLC	2	105	cCRT→IT	TRT	PBC	A: Nivolumab B: nivolumab + ipilimumab	mPFS: A 25.8 mo, B 25.4 mo	G ≥ 2 Pne A 22.2%, B 29.4%.
COAST (NCT03822351)^[19]^	Stage III NSCLC	2	189	cCRT→HT	TRT	PBC	Durvalumab *vs*. durvalumab + oleclumab vs. durvalumab +monalizumab	ORR: D 17.9%, D + O 30.0%, D + M 35.5%; mPFS: D 6.3 mo, D + O NR, D + M 15.1 mo	G3-4 Pne D 9.1 %, D + O 6.8%, D + M 1.6%; G3-4 RP D 1.5% D + O 0, D + M 0
LuCa-MERIT-1 (NCT05142189) Cohort 5^[22]^	Stage III NSCLC	1	20	cCRT→HT	TRT	PBC	BNT116 + cemiplimab	1y EFS% 72.2% 1y OS% 95%	G1-2 Pneu 15% G3 pneu 10%
PACIFIC-6 (NCT03693300)^[24]^	Stage III NSCLC	2	117	sCRT→HT	TRT	PBC	Durvalumab	mPFS: 10.9 mo mOS 25.0 mo	G3-4 Pne 1.7%
KEYNOTE-799 (NCT03631784)^[25]^	Stage III NSCLC	2	216	cCRT + IT→HT	TRT	Cohort A paclitaxel + carboplatin Cohort B pemetrexed + cisplatin	Pembrolizumab	mPFS: A: 30.6 mo B: NA	G3-5 Pne A: 8.0% B: 6.9%
PACIFIC-2 (NCT03519971)^[169]^	Stage III NSCLC	3	327 (219:108)	cCRT + IT→IT *vs*. cCRT	TRT	PBC	Durvalumab *vs*. palcebo	mPFS: 13.8 mo *vs*. 9.4 mo mOS: 36.4 mo *vs*. 29.5 mo	G ≥ 3 RP or Pne 4.6% *vs*. 5.6%
EA5181 (NCT04092283)^[26]^	Stage III NSCLC	3	662 (335: 327)	cCRT + IT→HT *vs*. cCRT→IT	TRT	PBC	Durvalumab	mPFS: 15.5 mo *vs*. 16.8 mo mOS: 41.5 mo *vs*. 39.4 mo	G ≥ 3 Pne 6.6% *vs*. 4.8%
CheckMate73L (NCT04026412)^[27]^	Stage III NSCLC	3	925 (287: 320: 318)	cCRT + IT→IT *vs*. cCRT→IT	TRT	PBC	A: Nivo + cCRT/Nivo + Ipi B: Nivo + cCRT/Nivo C: cCRT/Durva	PFS: A 16.82 mo *vs*. B 1 7.38 mo *vs*. C 1 5.64 mo OS: A 34.6 mo *vs*. B 39.49 mo *vs*. C 39.79 mo	SAE Immune-mediated lung disease 4.61% *vs*. 3.14% *vs*. 0.63%
NICOLAS (NCT02434081)^[29]^	Stage III NSCLC	2	79	cCRT + IT→IT	TRT	PBC	Nivolumab	mPFS 12.7 mo mOS 38.8 mo	11.7% G3-5 Pne
DETERRED (NCT02525757)^[23]^	Stage III NSCLC	2	40	P1: cCRT→IT P2: cCRT + IT→IT	TRT	Paclitaxel + carboplatin	Atezolizumab	mPFS: P1 18.9 mo, P2 15.1 mo mOS: P1 26.5 mo, P2 NR	UK
NCT04905316^[170]^	LA-NSCLC	1/2	41	cCRT + IT→IT	TRT	PBC	Canakinumab→ canakinumab + durvalumab	2y PFS% 67%	NA
NCT03663166	Stage III NSCLC	1/2	19	cCRT + IT→IT	TRT	PBC	Ipilimumab→Nivolumab	1-y PFS 58%	Unaccepted AEs 6/19 (31.6%) Pne 21.05%
NCT02621398^[171]^	Stage III NSCLC	1	23	cCRT + IT→IT	TRT	Paclitaxel and carboplatin	Pembrolizumab	mPFS 18.7 mo mOS 29.4 mo	G ≥ 2 AE 67% G ≥ 2 Pne 33%
AFT-16 (NCT03102242)^[172]^	Stage IIIA/B NSCLC	2	62	Induction IT→cCRT + IT → IT	TRT	Paclitaxel and carboplatin	Atezolizumab	mPFS 30.0 mo	G ≥ 3 Pne 6.4%
APOLO (NCT04776447)^[30]^	Stage III NSCLC	2	32	Induction IT + CT→cCRT →IT	TRT	Paclitaxel and carboplatin	Atezolizumab	PP set: 1 yPFS 78.1 %	UK
PACIFIC-BRAZIL (NCT04230408)	Stage III NSCLC	2	49	Induction IT + CT → cCRT + IT→IT	TRT	Paclitaxel and carboplatin	Durvalumab	1yPFS 68.1 %	≥ G3 Pne 14%
SPRINT (NCT03523702)^[32]^	Stage III NSCLC	2	25	Induction IT→ cCRT + IT → IT	accelerated, - dose-painted RT		Pembrolizumab	PFS 26 mo	≥ G3 Pne 4%
NCT04003246	Stage III NSCLC	2	10	Concurrent IT + RT→IT	TRT -		Durvalumab	mPFS 208 d	SAE: Pne 30.0%
NCT04249362 (DUART)	Stage III NSCLC ineligible for CT	2	102	RT HT	Cohort A - standard radiotherapy Cohort B palliative radiotherapy (40 to < 54 Gy)		Durvalumab	Cohort A: mPFS 10.3 mo Cohort B: mPFS 7.6 mo	G ≥ 3 PRAE 9.8%
START-NEW-ERA (NCT05291780)^[161]^	LA-NSCLC	NA	35	SABR + CT→IT	SABR (35-40 Gy in 5 fractions)	UK	UK	mPFS: 34 mo mOS: 72 mo	Acute Pne 28%, ≥ G3 Pneu 6% Late Pne 11 %
DOLPHIN (jRCT2080224763)^[173]^	Stage III NSCLC	2	35	RT + IT→IT	TRT	NA	durvalumab 10 mg/kg every 2 weeks	mPFS 25.6 mo	G3-4 Pne or RP 11.8%

A comprehensive literature search was conducted in PubMed and ClinicalTrials. gov up to November, 2025. Additionally, ongoing trials and preliminary data presented at major international oncology conferences (ASCO 2025, ESMO 2025, WCLC 2025) were included to ensure timeliness. AE: adverse event; cCRT: concurrent chemoradiotherapy; CT: Chemotherapy; d: days; G: Grade; IT: immunotherapy; m: months; mOS: median overall survival; mPFS: median progression-free survival; NSCLC: non-small cell lung cancer; ORR: objective response rate; PBC: platinum-based chemotherapy; Pne: pneumonitis; PRAE: possible-related AEs; Pts: patients; RP: radiation pneumonitis; RT: radiotherapy; SABR: stereoractic ablative radiotherapy; sCRT: sequential chemoradiotherapy; TRT: thoracic radiotherapy; UK: unknown.

Multiple trials have evaluated PD-1/PD-L1 inhibitors maintenance after cCRT: pembrolizumab yielded a median progression-free survival (mPFS) of 18.7 months (LUN 14–179),^[14]^ whereas sugemalimab produced median PFS times of 15.7 months (GEMSTONE-301),^[15,16]^ respectively, suggesting that immunotherapy consolidation can improve mPFS time. Whether combining different drugs in consolidated immunotherapy results in better efficacy remains a pressing question, and a spectrum of immunotherapy-based strategies is under investigation. Dual checkpoint blockade has been explored: the randomized phase II SKYSCRAPER-03 study^[17]^ demonstrated that consolidation with the T cell immunoreceptor with Ig and ITIM domains (TIGIT) inhibitor tiragolumab plus atezolizumab yielded mPFS comparable to that of durvalumab alone (14.2 m *vs*. 13.8 m; HR 1.00; 95% CI, 0.84−1.19); BTCRC Lung16–081 showed that nivolumab or nivolumab + ipilimumab maintenance after cCRT was associated with mPFS of 25.8 and 25.4 months, respectively.^[18]^ Similar studies are also being conducted on the combination of different immunotherapy strategies. In the COAST study,^[19]^ durvalumab combined with either the CD-73 antibody (oleclumab) or anti-NKG2A (monalizumab) extended PFS compared to durvalumab alone (oleclumab: HR = 0.44; 95% CI, 0.26 – 0.75; monalizumab: HR = 0.42; 95% CI, 0.24 – 0.72). In the phase III R-ALPS trial, benmelstobart plus anlotinib has a tendency to reduce the risk of progression versus benmelstobart (mPFS 17.4 mo *vs*. 11.2 mo; HR 0.82; 95%CI, 0.63−1.08), but the difference was not statistically significant (*P* = 0.1218).^[20,21]^ The ongoing LuCa-MERIT-1 trial ^[22]^ is testing a mRNA therapeutic vaccine combined with cemiplimab to amplify systemic antitumor immunity. The above-mentioned research results confirm the significance of post-CRT ICIs maintenance therapy. However, it has not yet been proven that different drug combinations can comprehensively enhance clinical benefits. Regarding the R-ALPS study and COAST study, although it has improved PFS to a certain extent, we still need to await whether it can improve OS results, and further research is required.

Patients with stage III NSCLC who are medically unfit for cCRT frequently receive sCRT or, rarely, radiotherapy alone. Consolidation immune-checkpoint blockade is now being tested in this context. In the phase III GEMSTONE-301 trial, sugemalimab after sCRT reduced the risk of progression or death by 43 % relative to placebo, with median PFS 8.1 *vs*. 4.1 months (HR 0.57).^[15,16]^ PACIFIC-5 and PACIFIC-6 subsequently confirmed benefit with durvalumab administered after sCRT, yielding median PFS of 11.0 and 10.9 months, respectively.^[23,24]^ For the minority of patients unable to receive any chemotherapy, definitive radiotherapy (RT) followed by sequential immunotherapy is under prospective evaluation in the DUART and TRADE-hypo phase II studies.

#### cCRT with immunotherapy

Radiotherapy induces the release of tumor antigens; however, this effect diminishes rapidly shortly after the conclusion of radiotherapy. Starting immunotherapy within 2 weeks of radiotherapy may result in a better prognosis.^[8]^ Therefore, combining immunotherapy with cCRT to enhance their efficacy has become a popular topic in clinical research. Despite intense investigation, cCRT plus immunotherapy has yet to improve outcomes for locally advanced NSCLC. In the single-arm phase II KEYNOTE-799 trial, pembrolizumab given concomitantly with cCRT yielded a median PFS of 30.6 months in the carboplatin–paclitaxel cohort (A) and was not reached in the cisplatin–pemetrexed cohort (B).^[25]^ However, the randomized phase III PACIFIC-2 study^[169]^ added durvalumab to cCRT followed by 12 months of consolidation durvalumab or placebo and showed only a non-significant mPFS prolongation (13.8 mo *vs*. 9.4 mo; HR 0.85, 95%CI, 0.65–1.12). The EA5181 study^[26]^ further confirmed that durvalumab as concurrent and consolidative therapy with cCRT compared with consolidative therapy did not improve PFS (15.5 mo *vs*. 16.8 mo) or OS (41.5 mo *vs*. 39.4 mo). CheckMate 73 L,^[27]^ DETERRED^[28]^ and NICOLAS^[29]^ reported similarly negative efficacy signals. Unlike the PACIFIC paradigm—which enrolled patients who had already responded to cCRT, the current trials enroll treatment-naïve individuals who might with bulky tumors that are prone to early progression, factors that may attenuate efficacy. Moreover, radiotherapy-induced injury to innate immunity and the heightened toxicity of concurrent immune-chemoradiation can further compromise treatment delivery and antitumor activity.

#### Induction (Chemo) immunotherapy followed by consolidation radiotherapy

In clinical practice, for patients with stage III NSCLC have bulky tumors, chemotherapy combined with immunotherapy is administered first. The diminished lung lesions may have surgical opportunities or definitive radiotherapy may be administered. Induction ICIs plus chemotherapy followed by definitive RT or chemoradiotherapy (CRT) has become a kind of treatment modality. Across several phase II studies, induction ICIs±chemotherapy followed by (chemo) radiation and maintenance immunotherapy consistently produced median PFS values of 20–30 months. The APOLO study,^[30]^ atezolizumab plus chemotherapy, then cCRT, then consolidation atezolizumab, produced 20.8 months mPFS, while a single-arm study involving 75 patients received induction chemoimmunotherapy followed by CRT achieved 30.6 months mPFS and an unreached mOS.^[31]^ The AFT-16^[172]^ trial showed that upfront atezolizumab before cCRT yielded 30 months mPFS and a 2 years OS rate of 73.7 %. In PD-L1-high (TPS ≥ 50 %) patients, SPRINT demonstrated 26 months mPFS after pembrolizumab induction followed by individualized radiotherapy.^[32]^ A multi-institutional retrospective study^[33]^ enrolled 158 LA-NSCLC in China also provided preliminary evidence supporting the efficacy of induction immunochemotherapy followed by cCRT strategy. Collectively, these data support early immune priming as a strategy to improve objective response and extend disease control before definitive CRT. We speculate that the potential benefit mechanisms of this combined treatment strategy include the following points: chemotherapy-induced immunogenic cell death and ICI-mediated T-cell priming, maximizes synergistic systemic anti-tumor activity and achieves substantial tumor shrinkage; subsequent radiotherapy then eradicates resistant clones and further potentiates abscopal immunity, while markedly reducing tumor volume and radiation-field size leads to lower radiation-related toxicity; maintenance immunotherapy eradicates residual disease. These conceptual advantages await confirmation in phase III trials.

### LS-SCLC

The ADRIATIC study^[13]^ evaluated the consolidation efficacy of durvalumab and tremelimumab in stage I–III LS-SCLC that did not progress after cCRT. The mPFS and mOS in the durvalumab maintenance group was significantly higher than that in the placebo group (mPFS: 16.6 mo *vs*. 9.2 mo; HR = 0.76; 95% CI, 0.59–0.98; *P* = 0.02; mOS: 55.9 mo *vs*. 33.4 mo; HR = 0.73; 95% CI, 0.57–0.93; *P* = 0.01). However, not all maintenance immunotherapy has shown therapeutic benefits in LS-SCLC. The ACHILES study ^[34]^ of atezolizumab maintenance showed only a trend for PFS benefit (21.1 mo *vs*. 15.9 mo, *P* = 0.55), and STIMULI study^[35]^ also found no advantage for nivolumab plus ipilimumab over observation (mPFS 10.7 mo *vs*. 14.5 mo; *P* = 0.93). Additional randomized trials (GASTO-1052, ASTRUM-LC01) are ongoing to clarify the role of immune maintenance in LS-SCLC. The combination of different immunotherapy agents as maintenance strategy are also are also being carried out in LS-SCLC. The phase II trial ML41257 (NCT04308785) and AdvantTIG randomized phase II trial (NCT04952597), will evaluate the benefits of administering PD-1/PD-L1 antibody and TIGIT antibody combined or after CRT in patients with LS-SCLC. The KEYLYNK-013 randomized phase III trial (NCT04624204) will compare ICIs combined with poly ADP ribose polymerase inhibitor therapy strategy. There are also multiple ongoing studies on post-cCRT immune maintenance for LS-SCLC (Table 2 and Supplementary Table S2).

**Table 2:** Completed clinical trials of combined immunotherapy, radiotherapy, and chemotherapy strategies for LS-SCLC

Trial	Tumor	Phase	Pts	Design	RT	CT	IT	Outcome	AEs
ADRIATIC (NCT03703297) ^[13,174]^	LS-SCLC	3	730 (1:1:1)	cCRT→IT *vs*. cCRT	TRT	CE/PE	Durvalumab *vs*. Dur-valumab + Tremelim-umab *vs*. Placebo	PFS: Durvalumab 16.6 mo *vs*. placebo 9.2 mo OS: Durvalumab 55.9 mo *vs*. placebo 33.4 mo	≥ G3 Pne or RP 3.1% *vs*. 2.6%
ETOP/IFCT 4-12 STIMULI (NCT02046733)	LS-SCLC	2	153 (78 *vs*. 75)	cCRT→IT *vs*. cCRT	TRT	CE/PE	Nivolumab + ipilimumab	PFS: experimental 10.7 mo *vs*. observation 14.5 mo OS: experimental NR *vs*. observation 32.1 mo	≥ G3 Pne 9.0% *vs*. 1.3%
ASTRUM-LC01 (NCT05443646)	LS-SCLC	2	55	cCRT→IT	Hypofractionated Radiotherapy	CE/PE	Serplulimab	1yPFS 71.8%	≥ G3 Pne 5.45%
GASTO-1052 (NCT04418648)	LS-SCLC	2	98	cCRT→IT *vs*. cCRT	TRT	CE/PE	Toripalimab *vs*. observation	PFS: NR *vs*. 14.1 mo OS: NR *vs*. 30.3 mo	≥ G3 RP 0 *vs*. 2.0% ≥ G3 Pne 2.1% *vs*. 0
ACHILES (NCT03540420)^[34]^	LS-SCLC	2	179	cCRT→IT *vs*. cCRT	TRT	CE/PE	Atezolizumab *vs*. observation	PFS: 21.1 mo *vs*. 15.9 mo OS: 43.4 mo *vs*. 38.8 mo	≥ G3 RP 1.2% *vs*. 2.3% ≥G3 Pne 1.2% G5 Pne 2.3%
NA^[175]^	LS-SCLC	1/2	40	cCRT+IT→IT	TRT	CE/PE	Pembroli-zumab	PFS: 19.7 mo, OS 39.5 mo	≥ G2 Pne 15%
NCT03585998^[176]^	LS-SCLC	2	51	cCRT+IT→IT	TRT	CE	Durvalumab	PFS: 14.4 mo OS: NR	≥ G3 RP 2%
NCT04952597	LS-SCLC	2	126	cCRT+IT→IT	TRT	CE/PE	Arm A: Ociperlimab + Tislelizumab/ArmB: Tislelizumab ArmC: none	PFS: Arm A 12.6 mo; Arm B 13.2 mo; Arm C 9.5 mo	SAE: Arm A 60.98%, Arm B 47.62%, Arm C 27.91%
NRG-LU 005 (NCT03811002)^[177]^	LS-SCLC	3	544 (274 *vs*. 270)	cCRT+IT→IT *vs*. cCRT	3D-CRT (three-Dimensional Conformal Radiation Therapy)	CE/PE	Atezolizumab	PFS: 12 mo *vs*. 11.5 mo OS: 33.1 mo *vs*. 39.5 mo	≥ G3 Pne 5.6% *vs*. 3.1%

A comprehensive literature search was conducted in PubMed and ClinicalTrials. gov up to November, 2025. Additionally, ongoing trials and preliminary data presented at major international oncology conferences (ASCO 2025, ESMO 2025, WCLC 2025) were included to ensure timeliness. AE: adverse event; cCRT: concurrent chemoradiotherapy; CE: carboplatin-etoposide; CT: Chemotherapy; G: Grade; IT: immunotherapy; mOS: median overall survival; mPFS: median progression-free survival; NSCLC: non-small cell lung cancer; PE: cisplatin-etoposide; Pne: pneumonitis; Pts: patients; RP: radiation pneumonitis; RT: radiotherapy; sCRT: sequential chemoradiotherapy; TRT: thoracic radiotherapy; UK: unknown.

Ongoing trials are dissecting how timing and drug choice modulate efficacy of immune-chemoradiotherapy in LS-SCLC (Table 2 and Supplementary Table S2). NRG-LU005^[177]^ randomized patients to concurrent cCRT plus atezolizumab versus cCRT alone; the experimental arm had a non-significantly shorter mOS (33.1 mo *vs*. 39.5 mo; HR 1.11, 95 % CI: 0.85–1.45). A single-arm study of cCRT with durvalumab reported mPFS of 14.4 months, whereas pembrolizumab plus cCRT achieved an objective response rate (ORR) of 79 % and mPFS of 19.7 months with acceptable toxicity.^[175,176]^ The above research results indicate that the concurrent ICI-cCRT regimen in LS-SCLC does not seem to show clear benefits, which is consistent with the research results of LA-NSCLC.

## Synergistic anti-tumor mechanisms of radiotherapy, chemotherapy, and immunotherapy

To advance the development and clinical implementation of combination therapy strategies, a better understanding of the synergistic anti-tumor mechanisms of radiotherapy, chemotherapy, and immunotherapy in stage III lung cancer is required (Figure 2). The reconstruction of both the tumor immune microenvironment and the systemic anti-tumor immune microenvironment require a focus not only on the tumor’s local immune environment but also on the body’s overall systemic immune environment.^[36,37]^

**Figure 2:**
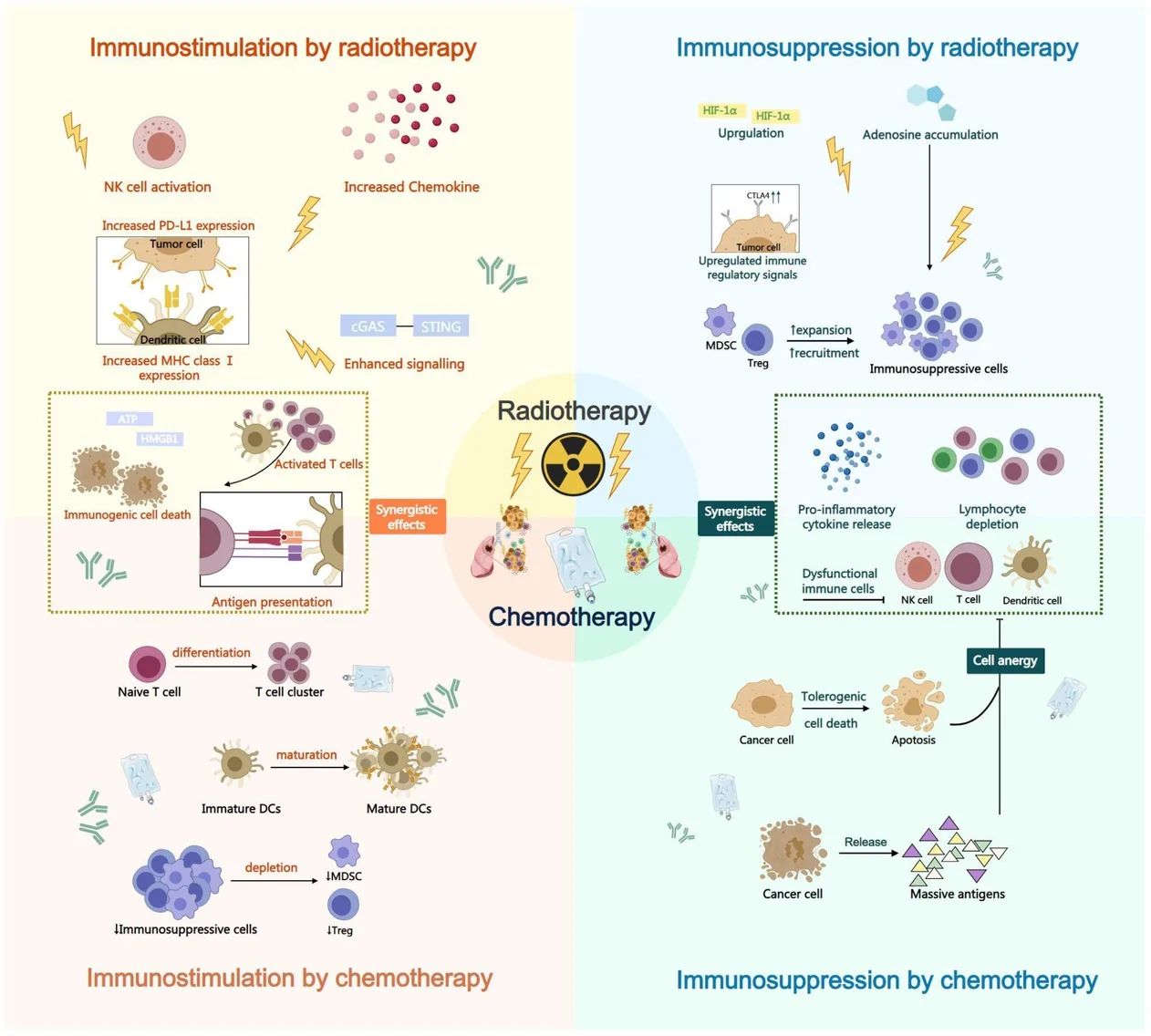
Bidirectional regulation of the anti-tumor immune efficacy by radiotherapy and chemotherapy.

### Bidirectional regulation of the anti-tumor immune efficacy by radiotherapy

#### The positive immunomodulatory anti-tumor effect caused by radiotherapy

From a molecular perspective, radiotherapy exerts cytotoxic effects primarily through direct DNA damage and reactive oxygen species (ROS)-mediated injury, leading to cell cycle arrest or cell death, thereby reducing tumor burden.^[38]^-Multiple mechanisms may lead to the immune regulatory effects of radiotherapy:^[39, 40, 41, 42, 43]^ immunogenic cell death (ICD) releases tumor antigens and enhances immunogenic gene expression;^[44,45]^ an inflammatory microenvironment is created through the release of ATP, HMGB1, calreticulin, and recruitment of inflammatory cytokines;^[44,46,47,48,49]^ local radiotherapy stimulates antigen release and antigen-presenting cells (APCs) maturation, enhancing dendritic cell antigen presentation;^[50, 51, 52, 53]^ chemokine expression (*e.g*., CXCL9, CXCL10) is induced, promoting T cell chemotaxis and activation;^[54,55]^ major histocompatibility complex (MHC) class I expression is upregulated, facilitating CD8^+^ T cell recognition;^[56]^ PD-L1 expression is increased, supporting T cell priming;^[57]^ NK cell activation is enhanced *via* upregulation of NK cell-activating ligands;^[58, 59, 60]^ radiotherapy can achieve abscopal effects through the intact cGAS-STING pathway and IFN-I signaling.^[61,62]^ These effects largely reflect the ability of radiotherapy to enhance downstream antigenicity and adjuvanticity of cancer cells. Importantly, many of these pathways can be enhanced by immunotherapy to improve the efficacy of radiotherapy, which has been explored in numerous clinical trials. In the PEMBRO-RT trial (NCT02492568),^[63]^ Stereotactic body radiotherapy (SBRT) enhanced the clinical efficacy of pembrolizumab by increasing the objective response rate from 18% to 36%. Further analysis with baseline biopsy samples^[64]^ showed a significant increase in immune cell density after treatment with anti-PD-1 alone or combined with radiotherapy.

#### The negative immunomodulatory anti-tumor effect caused by radiotherapy

Radiotherapy also shows negative effects on immune regulation. Irradiation of the bone marrow and lymphoid organs leads to lymphocyte depletion^[65, 66, 67]^ and the cytotoxicity of conventional fractionated radiotherapy is sufficient to deplete the migrating immune effector cells. Upregulation of negative immune regulatory signals, such as PD-L1 and CTLA-4, in tumors reduces tumor antigen-related immune responses and enhances immune escape functions. Radiotherapy increases the release of pro-inflammatory cytokines within tissues, such as chemokines CCL2, CCL5, and transforming growth factor β (TGFβ);^[68, 69, 70]^ promotes the recruitment and expansion of immunosuppressive cells,^[71,72]^ including regulatory T cells (Tregs) and cancer-associated fibroblasts; increases myeloid-derived suppressor cells (MDSCs); and enhances M2 tumor-associated macrophages to enhance the immunosuppressive effects. Consequently, the activities of CD8^+^ T and NK cells are inhibited. Radiotherapy-induced hypoxia upregulates hypoxia-inducible factor 1α, which supports the development of immunosuppressive cells, impairs dendritic cell function, and regulates immune checkpoint inhibitors like PD-L1.^[73]^ Furthermore, radiotherapy can cause extracellular adenosine accumulation, creating an immunosuppressive microenvironment that fosters tumor growth.^[74, 75, 76]^

#### The dose- and time-dependent impact of radiotherapy on immune enhancement

The efficacy of radiotherapy in tumors sensitive to anti-tumor immunity is influenced by the balance between immunostimulatory and immunosuppressive signaling pathways, with the dosing and timing of this potentiation being crucial.^[77]^ Different radiation doses and fractionation schemes appear to differentially regulate the immune microenvironment.^[40]^ SABR is believed to kill tumors more effectively, release antigens, induce immune responses, and reduce immune suppression.^[78,79]^ Low-fractionation schemes were more effective in inducing immune infiltration and abscopal effects compared with single ablative doses. Accelerated hyperfractionated radiotherapy appears to have better immune efficacy. The cytotoxicity of repeated daily conventional fractionated radiotherapy is sufficient to deplete the migrating immune effector cells.^[65, 66, 67]^ The immunostimulatory by radiotherapy can persist for a limited period of time after radiotherapy. Animal model studies have shown that, after 20 Gy of radiotherapy, the infiltration of CD8^+^ T cells into tumors begins to increase on day 3 and peaks on day 14. Additionally, radiotherapy increases antigen release and TCR diversity, with TCR clone diversity rising from day 4, though no clonal expansion of cytotoxic T lymphocytes was observed.^[80]^ By day 10 post-radiotherapy, CD8^+^ T cell exhaustion had begun to increase, suggesting cellular exhaustion and lymphocyte functional decline after radiotherapy. At 6 weeks post-treatment in the PEMBRORT trial,^[63,64]^ both helper and cytotoxic T cells increased, with regulatory T cells increasing significantly in the immunotherapy combined with SBRT group.

### Bidirectional regulation of the anti-tumor immune efficacy by chemotherapy

#### The positive immunomodulatory anti-tumor effect caused by chemotherapy

The synergistic effects of chemotherapy and immunotherapy have been clearly demonstrated in preoperative neoadjuvant treatments. After neoadjuvant treatment with nivolumab alone, 15% achieved complete pathological remission (PCR).^[81]^ In the CheckMate 816 and CheckMate 77T studies,^[82,83]^ neoadjuvant chemotherapy combined with nivolumab resulted in PCR rates of 24.7% and 25.3%, respectively, highlighting the synergistic effects of chemotherapy and immunotherapy.

Chemotherapy enhances anti-tumor immune responses *via* multiple mechanisms.^[84, 85, 86]^ Chemotherapy induces ICD and releasing damage-associated molecular patterns (DAMPs) that promote dendritic cell maturation and cytotoxic T lymphocyte activation.^[87,88]^ Chemotherapeutic agents increase tumor cell expression of HLA molecules and new antigens, enhancing anti-tumor immunotherapy.^[89]^ Chemotherapy may induce T cell differentiation into tumor-aggressive cytotoxic T cells. Specific drugs like cisplatin and carboplatin downregulate programmed death-ligand 2, improving T cell recognition.^[90,91]^ Taxanes increase MHC class II expression, promote dendritic cell maturation, enhance CD4^+^ and CD8^+^ functions, reduce Tregs, and decrease MDSCs.^[92, 93, 94]^ Pemetrexed selectively activates IFN-γ-producing NK cells and CD45RO+ memory T cells, increasing T cell infiltration and antigen presentation.^[95, 96, 97]^ Additionally, cytotoxic chemotherapies can deplete immunosuppressive cells, with platinum-based drugs significantly reducing MDSCs and cyclophosphamide decreasing Tregs.^[98]^

#### The negative immunomodulatory anti-tumor effect caused by chemotherapy

Chemotherapy also has immunosuppressive effects.^[99]^ It can lead to lymphodepletion with detrimental consequences, impair the proliferative and/or effector functions of peripheral T cells, influence the functionality of NK cells and other immune cells, and dampen the production of pro-inflammatory cytokines and chemokines. Concurrently, chemotherapy-induced tumor cell death often proceeds through apoptosis, a process that is generally regarded as non-immunogenic or tolerogenic. Yet, the sudden release of tumor antigens following chemotherapy can inundate the immune system, culminating in a state of immunosuppression and the induction of tolerance.

## Research on immune-related biomarkers for combined therapy

Although combined CRT and immunotherapy play a crucial role in the treatment of locally advanced unresectable lung cancer, there are still some key clinical research questions that need to be answered, including: (1) How do patients benefit from immunotherapy?; (2) How can the enhancement effect of radiotherapy on immunotherapy be evaluated?; (3) How can the differences between various combined treatment strategies be compared?; and (4) How can patients at high risk of adverse reactions to immunotherapy be identified? Therefore, effective biomarkers are required to better evaluate the state of the body and tumor microenvironment during or after CRT,^[66,100]^ in order to indicate clinical applications and research value in addressing these questions (Figure 3).

**Figure 3:**
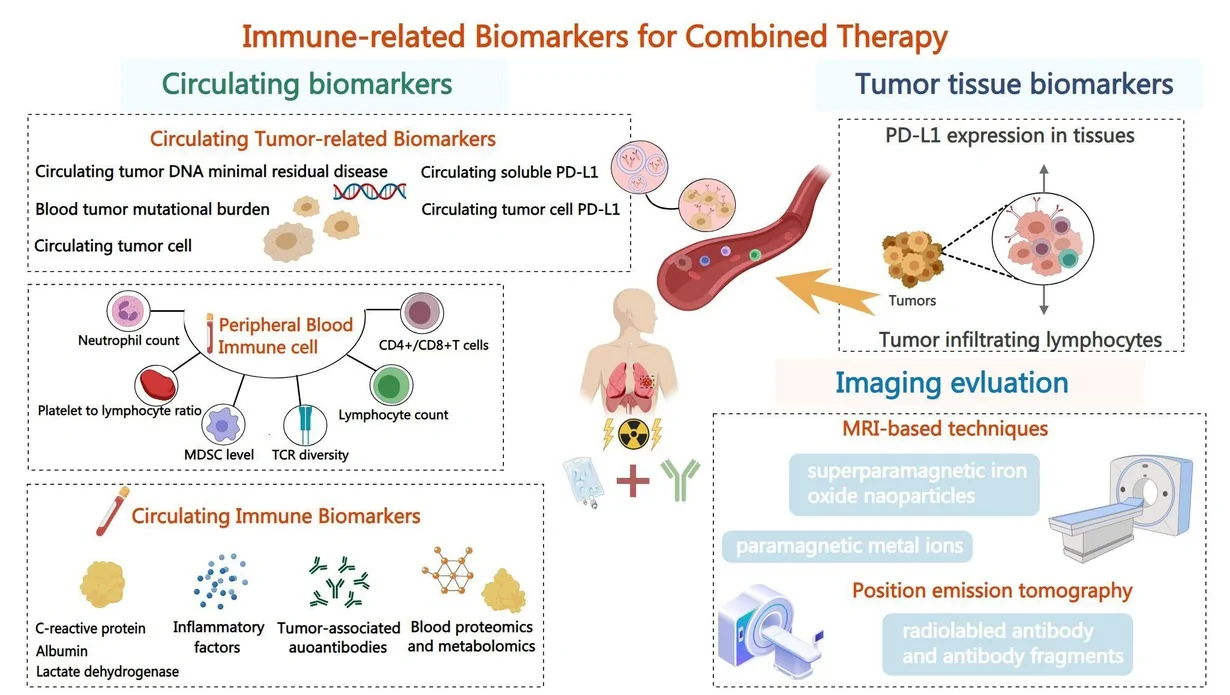
Immune-related biomarkers for anti-tumor immunotherapy, radiotherapy, and chemotherapy combined therapy.

### Biomarkers to evaluate the tumor microenvironment and immune status

#### Tumor PD-L1 expression

The tissue expression of PD-L1 is one of the most effective predictive biomarkers for the efficacy of immunotherapy. ^[1]^ In the PACIFIC study, patients with tumor cell (TC) PD-L1 ≥ 1% benefited from immunotherapy maintenance in terms of both PFS and OS.^[8,9]^ As PD-L1 expression is primarily based on tissue pathological testing, its expression exhibits temporal and spatial heterogeneity. Radiotherapy is believed to upregulate PD-L1 expression. The inability to dynamically assess tissue PD-L1 expression levels significantly limits the real-time prediction of treatment efficacy based on this biomarker.^[101]^

#### Evaluation of peripheral blood immune cells

Circulating peripheral blood immune cells are emerging as indicators of radiotherapy efficacy. Poor prognostic markers include radiotherapy-induced lymphopenia, elevated neutrophil counts prior to treatment, and a higher platelet-to-lymphocyte ratio.^[102, 103, 104]^ A high baseline absolute lymphocyte count (ALC) suggests a potential positive correlation between the efficacy of combined immunotherapy and radiotherapy,^[105]^ while nadir ALC is linked to reduced OS.^[106, 107, 108]^ Peripheral blood CD4^+^ T cells are also associated with prognosis,^[109]^ with an increased CD4^+^: CD8^+^ T cell ratio correlating with higher treatment efficacy in prostate cancer.^[110]^ Conversely, a lower CD4^+^: CD8^+^ ratio, increased CD8^+^ T cells, and higher PD-1+ CD8^+^ T cell counts relative to CD4^+^ T cells are linked to enhanced clinical benefits of combined immunotherapy and radiotherapy.^[111]^ Eosinophils are related to immunotherapy efficacy but also to immune-related adverse events.^[112]^ MDSC levels correlate with the efficacy of immunotherapy maintenance after CRT.^[113]^ In head and neck tumors, cCRT increases peripheral CD8^+^ T effector cells, MDSCs, Tregs, and checkpoint receptor-expressing T cells, particularly PD-1+ T cells.^[114]^

Evaluating T-cell clonal diversity is valuable in radiotherapy-induced CTLA-4 blockade treatment.^[115]^ Post-radiotherapy, dynamic changes in T-cell clones occur, with contracted clones and upregulated specific antigens.^[115]^ In patients with head and neck tumors, the total TCR count decreases post-radiotherapy; however, unique TCR sequences emerge, potentially related to the post-treatment response.^[114]^ A clinical study of patients with melanoma treated with ICIs showed that responders had higher TCR clonality in tumors before treatment than non-responders.^[116]^ In a study on neoadjuvant immunotherapy in patients with NSCLC,^[117]^ the total frequency of the top 1% of clones in the tumor repertoire was higher in responders than in non-responders. A study using sorted PD-1+ CD8^+^ T cells for TCR sequencing found that both high TCR diversity before immune checkpoint blockade (ICB) treatment and increased TCR clonality after ICB treatment were correlated with better treatment outcomes and prolonged PFS.^[118]^ In patients with cervical cancer undergoing cCRT, TCR diversity decreases post-cCRT, with a better prognosis observed in patients with high TCR diversity before cCRT.^[119]^

#### Peripheral blood tumor-related biomarkers

Currently, one of the most commonly used circulating biomarkers post-CRT is circulating tumor DNA (ctDNA) in LA-NSCLC.^[98]^ Previous study showed that, in the group receiving only cCRT, the 24-month freedom from progression was 0% and 100% for patients with and without detectable ctDNA minimal residual disease (MRD) after CRT, respectively.^[120]^ Early clearance of ctDNA during radiotherapy translates into significantly longer mPFS.^[121]^ This kinetic pattern is reproducible across multiple cohorts.^[122]^ These study also found that patients with detectable ctDNA after cCRT benefit most from sequential immune checkpoint inhibitor (ICI) consolidation therapy. Similarly, LS-SCLC patients with undetectable ctDNA after cCRT had good outcomes with or without consolidation ICI, whereas ctDNA-positive post-radiotherapy patients derived survival benefit from consolidation ICI.^[123]^

In addition, blood TMB (bTMB) has only been evaluated as a predictive biomarker for ICI responsiveness in treatment-naive patients with SCLC.^[124,125]^ The bTMB quantification technique was the same as that used to demonstrate the predictive value of bTMB for PFS in patients with NSCLC receiving atezolizumab for recurrence.^[126]^ Nonetheless, challenges such as variability in assay results, tumor heterogeneity, and the absence of standardized diagnostic thresholds can affect its reliability and consistent application in patient care, thereby constraining the broad adoption of this promising technology.

Additionally, circulating tumor cells have applications in evaluating the efficacy of anti-tumor treatment. In a study involving 38 patients, autophagy-activated circulating tumor cells were associated with shorter event-free survival (EFS) and OS following cCRT.^[127]^ Some studies have also suggested that circulating tumor cell PD-L1 testing and peripheral blood-soluble PD-L1 testing could potentially supplement measures of tissue PD-L1 expression.^[128, 129, 130]^

#### Circulating immune biomarkers

High C-reactive protein, low albumin, and high lactate dehydrogenase levels are associated with a poor prognosis in immunotherapy.^[100]^ Radiotherapy can promote the regulation of various inflammatory factors, such as interferons, IL-4, IL-5, TGFβ, IL-1β, IL-6, and tumor necrosis factor. Among these, IL-6 and IL-8 levels were associated with patient prognosis.^[131,132]^

Circulating tumor-associated autoantibodies are induced by tumor autoantigens, leading to a humoral immune response.^[133,134]^ Currently, tumor-associated autoantibodies appear to be correlated with tumor prognosis, particularly in the context of immunotherapy.^[135,136]^ However, there are currently rare validation studies on post-CRT.

Peripheral blood proteomics and metabolomics have also become popular research topics in recent year.^[137,138]^ A study conducted proteomic and metabolomic analyses of plasma at different time points after SBRT in patients with prostate cancer^[139]^ showed that the DNA damage response increased within the first hour of treatment and returned to baseline levels after 1 month.

### Imaging evaluation of combined therapy

In the field of imaging evaluation of immunotherapy, different systems based on conventional imaging, such as the immune-Response Evaluation Criteria in Solid Tumors (iRECEIST) and immune-modified Response Evaluation Criteria In Solid Tumors (imRECIST),^[140,141]^ are currently in use. However, whereas evaluating radiotherapy is relatively straightforward, assessing the subsequent functional efficacy of immunotherapy poses certain limitations. This is mainly due to inflammation and immune responses manifesting within the lesions after radiotherapy.

Magnetic resonance imaging (MRI) based techniques is a powerful tool for clinical imaging, which might be useful for evaluating the immune response. Currently, paramagnetic metal ions, such as gadolinium and manganese, are used to detect inflammatory responses in atherosclerosis.^[142]^ Some special contrast agents, such as superparamagnetic iron oxide nanoparticles,^[143]^ can be phagocytosed by macrophages and monocytes, and are used to assess certain types of inflammation, such as infections, atherosclerosis, and irritable bowel disease. In animal models of breast cancer,^[144]^ these agents induce dynamic changes in tumor-associated macrophages, and may serve as a new biomarker for the evaluation of immune-targeted therapies.^[145]^ However, owing to their low resolution and immature technology, these methods are still in the research stage.

Positron emission tomography (PET) is a promising imaging technique for imaging the immune response. However, 18F-fluorodeoxyglucose (FDG) is not specific to any particular leukocyte population, leading to issues with background signals, especially in areas of high glucose utilization (such as the brain, heart, or FDG-avid tumors) or near hypoxic regions.^[100]^ In oncology, this problem is further complicated by the phenomenon of pseudo-progression,^[146]^ wherein it is difficult to distinguish between increased immune activity after successful treatment and increased tumor metabolism after treatment failure. Increasing preclinical data suggests that radiolabeled antibody and antibody fragments can be used to image specific leukocyte populations,^[147, 148, 149, 150]^ potentially overcoming some of the limitations of the existing PET tracers. Additionally, integration of ctDNA and PET/CT metabolic parameters refine outcome prediction in LA-NSCLC: undetectable post-CRT ctDNA plus absolute change of metabolic tumor volume is associated with markedly prolonged PFS and OS,^[151]^ underscoring their potential for response-adapted treatment strategies and for identifying patients who may forgo ICI consolidation.

## Unresolved clinical issues and perspective for locally advanced unresectable lung cancer

### Multidisciplinary treatment strategy for locally advanced lung cancer

With the continuous advancement of neoadjuvant immunotherapy in early-stage NSCLC,^[82,152,153]^ the concept of perioperative neoadjuvant immunotherapy has become deeply ingrained in clinical research and practice. Neoadjuvant immunotherapy can significantly reduce the tumor size and downstage the clinical stages of tumors. Consequently, some tumors that are initially deemed inoperable may become resectable after neoadjuvant treatment. This development has blurred the definition of unresectable NSCLC, making some patients surgical candidates. For patients who cannot undergo surgery after neoadjuvant treatment, local radiotherapy may be added, potentially missing the optimal timing for cCRT. Therefore, developing the best treatment strategy for these patients is a pressing clinical issue. Additionally, for LA-NSCLC patients with epidermal growth factor receptor (*EGFR*) mutations, immunotherapy maintenance showed suboptimal efficacy in the PACIFIC subgroup analysis.^[9]^ The LAURA study^[154]^ included 216 patients with *EGFR*-sensitive mutations and unresectable stage III NSCLC who had not progressed after definitive CRT. The mPFS was 39.1 months in the osimertinib group *versus* 5.6 months in the placebo group (*P* < 0.001; HR = 0.16). Therefore, the TKIs maintenance seems to offer an advantage for patients with *EGFR* mutations. Consolidation trials (NCT07235306, NCT05170204) are also underway evaluating targeted therapy after cCRT in patients with unresectable LA-NSCLC harboring anaplastic lymphoma kinase (ALK) rearrangement and other actionable genomic alteration (AGA+). Additionally, trails (NCT05740943, NCT04636593, *et al*) are also exploring TKIs as induction therapy before definitive CRT in ALK-rearranged or EGFR-mutant LA-NSCLC. Developing personalized treatment strategies require a multidisciplinary collaboration.^[155]^

Owing to the inherently aggressive biology and propensity for early systemic dissemination of SCLC, the curative-intent treatment paradigm for LS-SCLC continues to be cCRT. Surgical resection is contemplated only in the < 5% of patients who present with peripherally located, pathologically confirmed T1–2N0M0 lesions. While pilot trials are exploring neoadjuvant chemo-immunotherapy followed by resection in highly selected cases, these protocols remain experimental and are restricted to patients with excellent performance status and minimal tumor burden.^[156]^ Consequently, cCRT—now increasingly coupled with consolidative ICIs or investigated in tandem with novel agents—remains the evidence-based cornerstone of curative therapy for LS-SCLC. However, up to 30 % of LS-SCLC patients present with bulky masses which cannot perform early-cCRT, and two-to-four cycles of induction chemotherapy can achieve sufficient tumor shrinkage to permit safe cCRT. This obligatory delay has created a therapeutic window in which ICIs can be added during induction without increasing pulmonary toxicity. Prospective single-arm and randomized trials (*e.g*., NCT04189094, NCT07055581, NCT07091305, NCT04539977, NCT05496166) are now formally testing induction chemotherapy plus ICIs followed by cCRT, with early safety data confirming feasibility and encouraging response rates; efficacy endpoints are pending.

Furthermore, approximately 20% of patients with unresectable stage III disease are unsuitable for CRT owing to poor physical condition, severe tumor symptoms, advanced age, or other factors. These patients are currently treated primarily with sCRT or radiotherapy alone or chemotherapy alone. Therefore, it is necessary to further adjust the treatment strategies for these patients.

In conclusion, the heterogeneity and complexity of both LA-NSCLC and LS-SCLC mandate multidisciplinary evaluation to devise individualized, evidence-based treatment plans (Figure 4).

**Figure 4:**
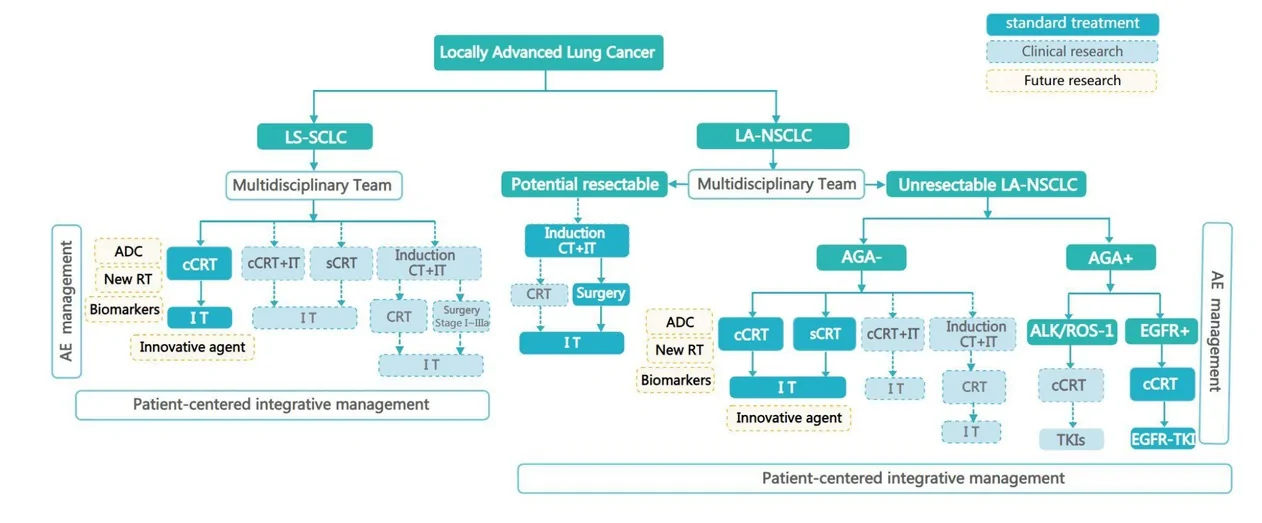
Multidisciplinary treatment strategy for locally advanced lung cancer.

### Combination and sequence of different treatments

The different strategies of combined RT, chemotherapy and immunotherapy are being actively explored (Figure 1). Current evidence establishes immune maintenance after cCRT as the standard-of-care for both LA-NSCLC and LS-SCLC, which offers high efficacy and a significantly improved prognosis. Adding immunotherapy concurrently with CRT has not demonstrated any improved efficacy in a series of studies, and concerns regarding safety in clinical studies pose challenges to its implementation in clinical practice.^[65]^ Given that radiation-induced immunosuppression may partly underlie treatment failure, future strategies should aim to minimize damage to the innate immune compartment and to pharmacologically modulate residual innate immunity for optimal antitumor efficacy. Sequential CRT and immunotherapy provide good patient tolerance; however, clinical research results indicate a suboptimal prognosis. In patients with bulky disease that precludes upfront cCRT or in whom surgical down-staging is sought, induction immunotherapy ± chemotherapy followed by definitive radiation achieves response rates and survival outcomes similar to standard post-cCRT immunotherapy maintenance.^[157]^ Although early signals with this strategy are encouraging, definitive efficacy must await confirmation in phase III randomized trials. Prospective randomized trials and real-world studies remain mandatory to confirm non-inferiority and optimal sequencing.

### Radiotherapy regimen

Different radiation doses and fractionation schemes appear to differentially regulate the immune microenvironment.^[40]^ SBRT, also known as stereotactic ablative radiotherapy (SABR), employs modern imaging and dose-delivery technologies to administer higher doses per fraction over fewer sessions. SBRT is believed to kill tumors more effectively, release antigens, induce immune responses, and reduce immune suppression.^[78,79]^ When compared with conventional fractionated radiotherapy (CFR), accelerated hyperfractionated radiotherapy appears to have better immune efficacy.^[40,158]^ Several studies^[159,160]^ combining CFR with SBRT for enrolled unresectable LA-NSCLC have shown clinical efficacy, and early safety data are acceptable, which requires prospective validation in adequately powered, randomized trials. Furthermore, START-NEW ERA^[161]^ is the first trial investigating the feasibility and efficacy of ChT-SAbR-IO in patients with unresectable LA-NSCLC. The median local control was not reached and the mPFS was 34 months, while only 3 (9%) discontinued IO due to toxicity. The SBRT-IO strategy should be further investigated in a phase III trial.

As to the SCLC, the considerations of radiotherapy have primarily focused on the radiotherapy fractionation regimen. Current evidence supports risk-stratified selection: hyper-fractionated 45 Gy or accelerated 54 Gy for fit patients seeking maximal survival;^[162]^ moderately hypofractionated 45 Gy/15 f for those needing lower acute toxicity without compromising efficacy;^[163]^ and 40–50 Gy/15–25 f for frail or resource-limited settings. Nevertheless, how divergent radiotherapy fractionation regimens shape the tumor immune microenvironment and consequently modulate the efficacy of immunotherapy remains essentially unknown. The STIMULI study^[35]^ suggested that twice-daily radiotherapy may be more beneficial for patients undergoing maintenance immunotherapy. Prospective randomized comparisons of these different radiotherapy fractionation regimens with post-CRT ICIs treatment are now the priority.

### The use of innovative anti-tumor drugs

Novel antitumor agents are being systematically explored in both LA-NSCLC and LS-SCLC, including PD-1/PD-L1 monoclonal antibodies combined with TIGIT or LAG-3 antibodies, as well as bispecific antibodies (*e.g*., anti-VEGF/PD-1) as maintenance therapy. However, further intensification of checkpoint blockade—whether through additional antibodies or checkpoint-centric combinations—has yielded only limited benefit, underscoring the need for alternative biological strategies. Recent breakthroughs in lung-cancer drug development (*e.g*. antibody–drug conjugates [ADCs], T-cell engagers) are generating renewed optimism for curative-intent treatment of limited-stage disease. ADCs have produced breakthroughs in metastatic NSCLC and encouraging signals in extensive-stage SCLC. Whether substituting ADC for conventional cytotoxic agents within curative-intent chemoradiotherapy can enhance therapeutic efficacy remains to be rigorously investigated. For SCLC, the DLL3-directed T-cell engager tarlatamab outperforms chemotherapy in refractory disease,^[164]^ and a post-cCRT maintenance trial has been initiated. Maintenance therapy with tarlatamab may elicit a more robust antitumor immune response and could represent a breakthrough in the treatment of LS-SCLC. Moreover, therapeutic cancer vaccines and cellular therapies also under investigation in stage III NSCLC.

### Toxicity and clinical management

Researchers and clinicians are concerned about the treatment related adverse events (TRAEs) in the combined treatment approach. Due to the complex collaborative mechanisms such as the radiation directed inflict cytotoxic effects on lung tissues, cross-reactivity between tumor and lung tissue antigens and ICI mediated inflammatory reactions, the incidence of pneumonia, including radiation, immune, and overlapping pneumonia, in patients undergoing CRT combined with immunotherapy is high.^[165]^ In the PACIFIC study,^[9]^ durvalumab raised any-grade pneumonitis/pneumonia rates (34% *vs*. 25 % and 13% *vs*. 8%), yet grade ≥ 3 events remained ≤ 4% and were not statistically increased. Real-world PACIFIC-R data show 9.5% discontinuation for pneumonitis/ILD. Notably, the concurrent approach tested in KEYNOTE-799 produced grade ≥ 3 pneumonia in 6%–8% of patients and fatal pneumonia in 1%–4%. Multivariable analyses ^[166]^ confirm that, after cCRT, ICI exposure, irradiated volume and cumulative dose independently predict grade ≥ 2 pulmonary toxicity, whereas the incremental risk of grade ≥ 3 injury is driven by dosimetric rather than immunologic factors. ICIs can trigger immune-related cardiac injury (myocarditis, conduction block, heart failure, coronary or pericardial disease), while thoracic radiotherapy independently produces overlapping toxicities. The mechanisms underlying combined immune radiotherapy-induced cardiovascular damage include vascular damage, epithelial cell injury, autoantigen release, increased antibodies, increased activation of immune cells, release of inflammatory factors, increased fibrosis, and metabolic abnormalities.^[167]^ Studies such as GEMSTONE-301 and KEYNOTE-799 have reported myocardial-related injuries,^[167]^ underscoring the need for prospective cardiac surveillance and mechanistic studies in combined immune-radiation protocols.

Minimizing TRAEs hinges on rigorous patient selection and the delivery of optimally treatment regimens, exemplified by the use of smaller, meticulously delineated radiation fields that maximize tumor dose while sparing normal lung parenchyma. Proactive strategies for the timely identification, accurate diagnosis, and effective management of TRAEs are essential to optimize patient outcomes. Clinical studies have attempted to evaluate the feasibility and effectiveness of mobile and wearable medical devices for improving the early detection of treatment-related adverse events (AEs) after cCRT and durvalumab consolidation in patients with unresectable LA-NSCLC (NCT04381494).

### Precision treatment guided by biomarkers

Biomarkers are likewise anticipated to refine patient selection for combined-modality therapy in local advanced lung cancer, especially for guiding post-CRT immunotherapy. Pre-CRT tissue samples may not accurately reflect the post-treatment immune environment. Unlike neoadjuvant therapy, irradiation leaves no surgical resection specimens or biopsy tumor samples for response assessment, and post-CRT tumor lesions confounds RECIST evaluation on routine CT. Whether ctDNA or ctDNA-based MRD can triage patients to consolidation ICI is therefore an active focus. Current research supports the clinical recommendation of this strategy, including the favorable prognosis for post-cCRT ctDNA-negative patients and the significant benefit of immune consolidation therapy for ctDNA-positive patients. However, heterogeneity in sampling time-points, sequencing platforms and algorithms, together with high cost and limited external validation, preclude implementation as a stand-alone test. Moreover, ctDNA only reflects tumor clearance and provides no information on immune activation. Complementary assays that quantify radiation-induced, antigen-specific T-cell expansion or functional cytolytic capacity are urgently needed to identify patients who have achieved sufficient systemic immunity and to spare others from unnecessary immunotoxicity. However, the assessment of the host’s immune response to treatment, including strategies such as TCR analysis, is still in the initial stage of exploration. There are adjustments needed in clinical validation and standardization of detection, as well as challenges in terms of clinical benefits and cost-effectiveness. Investigations have commenced on the development of integrated assessment strategies that incorporate diverse biomarker approaches, such as the overall assessment of liquid biopsy and PET/CT. Moreover, the integrating “patient-tissue-liquid-imaging” multimodal assessment of tumor tissue, liquid biopsy biomarkers, host immune response evaluation and imaging may be more beneficial to accurately identify patients most likely to benefit from specific combination therapies.

There is an urgent requirement for predictive models and biomarkers to predict immune related adverse events (irAEs) or radiation toxicities. While several candidates might associate with irAEs,^[168]^ the frequent linkage between low-grade irAEs and survival benefit precludes withholding immunotherapy on the basis of such signals. Conversely, no reliable markers exist for life-threatening events (*e.g*., severe pneumonitis or myocarditis). Radiation-induced thoracic injury and combined immunoradiation toxicities are governed less by biomarkers than by treatment-related variables (irradiated volume, cumulative dose, fractionation, and cardiac V30), necessitating integration of dosimetric, clinical, and molecular data. This multidimensional complexity underscores the need for multimodal datasets and artificial-intelligence models to build robust, prospective AE-prediction algorithms.

### Patient-centered integrative management

Although cCRT followed by ICIs consolidation is the evidence-based standard for LA-NSCLC and LS-SCLC, its real-world delivery is fraught with hurdles: (1) limited patient understanding of curative-intent radiotherapy, necessitating intensive counselling to secure adherence; (2) complex comprehensive treatment affects the patient’s social function and psychological state; (3) prolonged immunotherapy schedules that impose repeated medical visits and substantial socio-economic cost; (4) radiation related debilitating esophagitis, pneumonitis and dermatitis that impair nutrition and quality of life; (5) cumulative toxicities from chemotherapy and ICIs that generate both morbidity and health-care expenditure.

A genuinely patient-centred model therefore exploits advanced radiation technologies—smaller fields, shorter courses, and lower integral dose—to minimize acute injury while preserving tumour control. Future iterations will layer biomarker-driven precision, subcutaneous or depot immunotherapy to reduce clinic visits, and wearable sensors that provide longitudinal, real-time monitoring of symptoms, cardiopulmonary function, and treatment adherence. Rehabilitation exercises to restore the cardiopulmonary function and strengthening social and psychological support to improve the patient’s condition are also key points that need to be focused on in the overall treatment. Integrating these innovations into a “smart”, continuous-care ecosystem will transform multidisciplinary therapy from a protocol-centered prescription into a personalized, pragmatic, and tolerable journey for every individual.

## Conclusions

Concurrent CRT followed by immunotherapy maintenance therapy is a cornerstone for the treatment of unresectable LA-NSCLC and LS-SCLC, offering patients a more comprehensive approach to combat their disease. The evolution of treatment paradigms in lung cancer reflects a deepening understanding of tumor immunology and a commitment to personalized medicine. The dynamic landscape of lung cancer treatment is further shaped by emerging strategies that integrate biomarkers to offer non-invasive methods of assessing treatment response and guiding therapeutic decisions. There are significant challenges that need to be addressed to optimize this treatment approach and make it accessible to patients who may benefit.

## Supplementary Material

Supplementary Material Details
