# Supplementary material for: Anti-tumor immunotherapy, radiotherapy, and chemotherapy multimodal combination strategies and interactions in unresectable, locally advanced lung cancer: Clinical practice, mechanism, and biomarker research
Source: J Transl Int Med. 2026 Jun 13;14(5):699–718. doi: 10.1515/jtim-2026-0044 (PMC13726654; doi:10.1515/jtim-2026-0044)
Supplement: Supplementary file 1 [file jtim-2026-0044_sm.pdf]

Supplementary materials

Supplementary Table S1: Ongoing clinical trials of combined immunotherapy, radiotherapy, and chemotherapy strategies for unresectable, locally advanced NSCLC

Trial	Tumor	Phase	Pts	Design	RT	CT	IT	Outcome	Status
NCT04230408	Stage III NSCLC	2	48	Induction IT+CT→ + cCRT+IT → IT	TRT	carboplatin, paclitaxel	Durvalumab	12 m PFS rate	Completed
NCT04085250	Stage III NSCLC	2	264 Randomized	Induction IT+CT→cCRT →IT or observation	TRT	Docetaxel +Cisplatin	Nivolumab	PFS	Active, not recruiting

NCT04765709 (BRIDGE)	Large Volume Stage III NSCLC	2	10	Induction IT+CT→IT+RT →IT	TRT	PBC	Durvalumab	the proportion of patients who did not progress and eligible for immunotherapy plus radiotherapy	Active, not recruiting
NCT03589547	Stage III NSCLC	2	11	cCRT→ SBRT+IT	SBRT 20 Gy (2 fractions of 10 Gy)	UK	Durvalumab	No. pts ≥G2 AE	Active, not recruiting
NCT05557552	Elderly and/or Frail Stage III NSCLC	2	56	Induction IT+CT→ IT+RT→ IT	standard dose (60 Gy/30Fx) *vs* decreased thoracic radiotherapy 50 Gy/25Fx	PBC	anti-PD-1/L1	PFS	Recruiting
NCT05128630	Stage III NSCLC	2	45	Induction IT+CT→RT→	reduced-dose HypoFrx-RT		Durvalumab	≥G3 PRAEs possibly related	Recruiting

				IT				adverse events	
NCT04982549	Stage III NSCLC	2	35	cCRT+IT → IT	5 fractions/ wk for ~6 wk (±3 d) (Total 60 Gy)	PBC	Durvalumab	⩾G3 irAEs	Unknown status
NCT05940532	Stage III NSCLC	2	41	Induction IT+CT→RT or surgery→ IT	(MDT)-guided radiotherapy or surgery	PBC	Sugemalimab	ORR	Recruiting
NCT06031597	LA-NSCLC	3	105	Induction IT+CT Cohort A: cCRT+IT Cohort B: RT+IT Cohort C: RT→ IT	Volumetric-modulated arc therapy (VMAT) and image-guided radiotherapy (IGRT).	PBC	anti-PD-1/L1	radiation pneumonitis	Not yet recruiting
NCT03840902	Stage III NSCLC	2	153 Randomi	cCRT +IT (M7824) *vs*	Intensity Modulated Radiation Ther	PBC	M7824 Durvalumab	PFS	Terminated

			zed	cCRT → IT	apy (IMRT)				
NCT05696782	Stage III NSCLC	2	28	cCRT→ IT	TRT	PBC	Durvalumab	Number of Participants to Achieve Fidelity to Early Treatment with Durvalumab-Early Initiation (1–14 days)	Recruiting
NCT03379441	Stage III NSCLC	2	126	cCRT→IT	TRT	PBC	Pembrolizumab	OS	Unknown status
NCT04364048	Stage III NSCLC	2	10	Induction IT→cCRT→ IT	TRT	PBC	Durvalumab	PFS<br>AE	Terminated
NCT05468242	LA NSCLC	2	116	Induction CT+IT+Bev<br>cCRT+IT+Bev	TRT	PBC	Tislelizumab	PFS	Recruiting

				→ IT<br>Or induction CT+IT→cCRT+ IT→IT					
NCT04577638	LA-NSCLC	2	8	RT+IT→ IT	IMRT 66 Gray (Gy) /24 fractions of 2.75 Gy)	NA	Nivolumab	DCR	Completed
NCT04351256<br>(TRADE-hypo)	Stage III NSCLC<br>Unfit for Chemotherapy	2	88	RT+IT→IT	Thoracic Radiotherapy (TRT) 30 x 2 Gy (60 Gy)<br>(TRT) hypofractionated 55 Gy/20f of 2.75 Gy	NA	Durvalumab	Toxicity (pneumonitis)<br>ORR	Active, not recruiting
NCT04310020	Stage II/III NSCLC	2	47	RT→IT	Hypofractionated RT (15f)	NA	Atezolizumab	G3-5 AEs	Recruiting
NCT05136846	Stage II/III NSCLC	1	28	cCRT→IT	TRT	paclitaxel, carboplatin<br>Papaverine	Durvalumab	MTD of Papaverine	Recruiting
NCT053868	Stage III	2	65	cCRT+IT→ IT	TRT	PBC	GFH018	ORR	Comple

88	NSCLC						Toripalimab		ted
NCT06003075	Stage III NSCLC	2	37	Induction IT+CT→ cCRT or surgery→ IT	Surgery or TRT	PBC	Nivolumab	ORR	Terminated
NCT01993810	Stage II-IIIB NSCLC	3	330	cCRT→ IT	TRT *vs.* proton beam RT	PBC	Durvalumab	OS	Active, not recruiting
NCT05177497	Stage III NSCLC	2	19	cCRT→ IT	TRT	PBC	SHR-1701	AE	Unknown status
NCT05718297	ALK+ Stage III NSCLC	2	44	cCRT→ IT or ALK-TKIs	TRT	PBC	Durvalumab *vs* Brigatinib	PFS	Withdrawn
NCT04380636	Stage III NSCLC	3	870	cCRT+IT→ IT	TRT	PBC	Pembrolizumab plus Olaparib	PFS OS	Active, not

(MK-7339-012/ KEYLYNK-012)							*vs* pembrolizumab *vs* Durvalumab		recruiting
NCT06424899	Stage III NSCLC	92	92	Induction IT+CT→cCRT or Surgery→IT *vs.* cCRT+IT → IT	Surgery or TRT	PBC	Adebrelimab	2-y EFS%	Recruiting
NCT06102057	LA-NSCLC	2	110	cCRT+IT→ IT	TRT *vs* FDG-PET-based radiotherapy	PBC	durvalumab	AEs and TTP (Time to locoregional progression)	Recruiting
NCT06124118	LA-NSCLC	1	30	cCRT+IT→ IT TTFields	TRT	PBC	Durvalumab	DLTs	Recruiting
NCT03141359	LA-NSCLC	2	61	SBRT + cCRT→ IT	SBRT+ IMRT	PBC	Durvalumab	1-y PFS	Active, not

									recruiting
NCT04748419	Stage III NSCLC	1/2	6	cCRT→IT	TRT+hfRT	PBC	Durvalumab	AEs<br>PFS	Active, not recruiting
NCT05198830	Stage III NSCLC	2	42	cCRT→IT	TRT	PBC+TRC102 *vs*. PBC	Durvalumab	PFS	Recruiting
NCT04392505	Stage III NSCLC	2	100	cCRT→IT	TRT	PBC	Durvalumab	Tumor mutational burden (TMB)	Active, not recruiting
NCT04585490	Stage III NSCLC	3	48	cCRT→MRD+: CT+IT→IT<br>MRD-: IT	TRT	PBC	Durvalumab+ tremelimumab	Change in ctDNA Level Following Chemotherapy	Recruiting
NCT05624996	LA-NSCLC	3	474	cCRT→ IT	IGRT (image guided RT) *Vs*. SBRT and conventional	PBC	Durvalumab	OS, PFS	Recruiting

					IGRT				
NCT03999710	LA-NSCLC	2	67	RT+IT→IT	TRT	NA	Durvalumab	2-y PFS%	Active, not recruiting
NCT05298423 (MK-7684A-006/ KEYVIBE-006)	Stage III NSCLC	3	784	cCRT+IT→IT *vs.* cCRT→IT	TRT	PBC	MK-7684A *vs.* Durvalumab	PFS, OS	Active, not recruiting
NCT04013542	Stage II/III NSCLC	1	21	RT+IT→IT	TRT		nivolumab and ipilimumab	AEs	Active, not recruiting
NCT05306847	Stage II/III NSCLC	2	93	IT+TKIs→cCRT or surgery→IT	TRT	PBC	Sintilimab + Anlotinib	Surgery rate	Not yet recruitin

									g
NCT03801902	PD-L1 high (>= 50%) LA-NSCLC	1	48	RT+IT→IT	Accelerated Hypofractionated Radiation Therapy *vs*. TRT	NA	Durvalumab, ±monalizumab/oleclumab	AE	Completed
NCT04866017	Stage III NSCLC	3	63	IT+cCRT→IT	TRT	PBC	Ociperlimab + tislelizumab/ tislelizumab/ durvalumab	PFS	Terminated
NCT03916419	Stage II/III NSCLC	2	26	cCRT→IT	(MR)-guided RT	carboplatin + paclitaxel	durvalumab	DLTs Local/ Regional control rate	Completed
NCT05358691	Stage III NSCLC	1	24	cCRT+ AN0025 →IT+AN0025	TRT	PBC	AN0025 Durvalumab	ECOGPS	Withdrawn
NCT06484491	Stage III NSCLC	NA	87	Induction CT→cCRT→IT	Dose-escalated IMPT	PBC	Durvalumab	SAE	Not yet recruitin

									g
NCT06734702	LA-NSCLC	3	497	Induction CT+IT→cCRT →IT *vs.* cCRT→IT	HyRT *vs.* CFRT	PBC	Tislelizumab	PFS	Recruiting
NCT07086456	LA-NSCLC	2	80	cCRT+IT→IT	TRT	Paclitaxel +Cisplatin	Surufatinib and Tislelizumab	2y PFS%	Recruiting
NCT05758116	LA-NSCLC	2	20	RT or sCRT→IT	TRT	UK	Tislelizumab	PFS	Recruiting
NCT06865339	LA-NSCLC	2	76	Induction IT±CT→RT or CCRT→IT	TRT	PBC	Cemiplimab + Fianlimab	ORR	Recruiting
NCT06944470	Stage II-III NSCLC	2	76	Induction IT+CT→CRT or surgery→ IT	TRT or surgy	PBC	JS207	PCR rate	Not yet recruiting
NCT06998719	Stage III NSCLC	2	80	Induction IT+CT→	TRT or surg	PBC	Durvalumab	Resection rate	Not yet recruitin

				surgery→ IT Or CCRT→ IT					g
NCT07052669	Stage III NSCLC	3	311	cCRT→IT	HyRT *vs*. CFRT	PBC	Immunotherapy	PFS	Recruiting
NCT06667908	Stage III NSCLC	2	130	cCRT→IT	TRT	PBC+ JNJ-90301900	Immunotherapy	ORR	Recruiting
NCT05798663	NCT05798663	2	178	Induction IT →cCRT±IT→IT	TRT	PBC	Atezolizumab *vs*. atezolizumab + tiragolumab	PFS	Active, not recruiting
NCT05888402	LA-NSCLC	2	50	Induction CT+IT or CT →cCRT→IT	TRT	PBC	Toripalimab	PFS	Recruiting

Trails in ClinicalTrials.gov were screened by "Non-Small Cell Lung Cancer stage III or " in the condition section and "Radiotherapy or chemoradiotherapy or radiation" the intervention/treatment section. The interventional studies with immunotherapy were enrolled in the Supplementary Table 1. The table was lasted update by November 2025.

AE: adverse event; cCRT: concurrent chemoradiotherapy; CFRT: conventionally fractionated radiotherapy (CFRT); CT: Chemotherapy; hfRT: Hypofractionated Radiation (hfRT); PBC: platinum-based chemotherapy; TRT: thoracic radiotherapy; ORR: objective response rate; Pts: patients; RT: radiotherapy; IT: immunotherapy; mPFS: median progression-free survival; mOS: median overall survival; NSCLC: non-small cell lung cancer; G: Grade; pne: pneumonitis; RP: radiation pneumonitis; sCRT: sequential chemoradiotherapy; SAE: Severe adverse effects.

Supplementary Table S2: Ongoing clinical trials of combined immunotherapy, radiotherapy, and chemotherapy strategies for LS-SCLC

**Trial**	**Tumor**	**phase**	**Pts**	**Design**	**RT**	**CT**	**IT**	**Outcome**	**Status**
NCT05353257	LS-SCLC	3	482	cCRT+IT→IT *vs.* cCRT	TRT	CE/PE	Serplulimab	OS	Active, not recruiting
NCT06371482 Camel-01	LS-SCLC	2	58	cCRT+IT→IT	TRT	CE/PE	Durvalumab	ORR/TRAE	Recruiting
NCT05034133	LS-SCLC	2	20	CT+IT→RT	TRT	PE	Durvalumab	PFS	Unknown status
NCT04189094	LS-SCLC	2	140	Induction CT+IT →cCRT→IT *vs.*	TRT	PE	Sintilimab	PFS	Unknown status

				CT Induction →cCRT				
NCT05496166	LS-SCLC	3	348	Induction CT+IT →cCRT *vs.* surgery→IT	TRT	PE	Adebrelimab	PFS	Not yet recruiting
NCT06527898	LS-SCLC	1/2	40	cCRT→CT+IT→IT	Hypofractionated radiotherapy	CE/PE	Adebrelimab	PFS	Recruiting
NCT04602533 (DOLPHIN)	LS-SCLC	2	105	cCRT+IT →IT *vs* cCRT	TRT	CE/PE	Durvalumab	PFS	Active, not recruiting
NCT02402920	LS-SCLC	1	83	A: cCRT+IT→IT B: CT→IT+RT→IT	TRT	CE/PE	Pembrolizumab	AEs	Active, not recruiting
NCT06140407	LS-SCLC	2	20	cCRT+IT→IT	TRT	CE/PE	Pembrolizumab	PFS	Terminated
NCT04539977	LS-SCLC	2	40	Induction CT+IT→	TRT	CE	TQB2450	ORR	Recruiting

				Surgery *vs* RT→IT					
NCT05904015	LS-SCLC	2	80	cCRT+IT→IT	Hypofractionated radiotherapy / TRT	CE/PE	Envafolimab	2-year PFS rate	Not yet recruiting
NCT04691063	LS-SCLC	3	486	cCRT+IT→IT	TRT	CE	Adebrelimab *vs*. Placebo	OS	Unknown status
NCT06295926	LS-SCLC	2	96	cCRT+IT→IT	TRT	CE/PE	Serplulimab	PFS	Recruiting
NCT04624204 (MK 7339-013/ KEYLYNK-013)	LS-SCLC	3	672	cCRT+IT→IT	TRT	CE/PE	Pembrolizumab *vs*. Pembrolizumab+Olaparib *vs*. placebo	PFS, OS	Active, not recruiting
NCT06095583	LS-SCLC	3	756	cCRT→IT	TRT	CE/PE	A: Tifcemalimab + Toripalimab<br>B: Placebo + Toripalimab<br>C: Placebo	PFS, OS	Recruiting

NCT05942508	LS-SCLC	2	20	cCRT→IT	TRT	PBC	TQB2450 Injection + Anlotinib	AE, SAE	Recruiting
NCT05617963 (DURVALUNG)	frail LS-SCLC	2	550	cCRT or SCRT→IT	TRT	CE/PE	Durvalumab	PFS	Recruiting
NCT04308785	LS-SCLC	2	24	cCRT→IT	TRT	PBC	Atezolizumab+tiragolumab or placebo	PFS	Terminated
NCT05623267	LS-SCLC	2/3	346	cCRT or SCRT→IT	TRT	CE/PE	Sugemalimab	PFS	Recruiting
NCT06117774	LS-SCLC	3	400	cCRT→BiTE or Placebo	TRT	CE/PE	Tarlatamab	PFS, OS	Recruiting
NCT06789796	LS-SCLC	3	636	cCRT or SCRT→IT	TRT	CE/PE	Iparomlimab and tuvonralimab or QL1604	PFS, OS	Not yet recruiting
NCT06773910	LS-SCLC	2	250	cCRT →IT	TRT	CE/PE	BMS-986489 *vs.* durvalumab	OS	Recruiting
NCT04647357	LS-SCLC	2	60	cCRT →IT	TRT	CE/PE	SHR-1316	PFS	Unknown status

NCT07200154	LS-SCLC	2	39	cCRT+IT→IT	TRT	PBC	Adebrelimab	PFS	Not yet recruiting
NCT07103408	LS-SCLC	2	56	cCRT+IT→IT	TRT	CE/PE	Iparomlimab + tuvonralimab +thymosin alpha 1	PFS	Not yet recruiting
NCT07055581	LS-SCLC	2	100	Induction CT+IT →cCRT→IT	TRT	CE/PE	Durvalumab	PFS	Not yet recruiting
NCT06719700	LS-SCLC	2	47	cCRT+IT→IT	TRT	CE/PE	Toripalimab and Surufatinib	PFS	Recruiting
NCT06784206	LS-SCLC	2	30	cCRT→ IT	TRT+ HART	CE/PE	Adebrelimab	TRAE	Not yet recruiting
NCT07091305	LS-SCLC	2	28	Induction CT+IT →cCRT→IT	TRT	CE/PE	Iparomlimab + tuvonralimab	PFS	Active, not recruiting
NCT06773156	LS-SCLC	2	37	cCRT→ IT	TRT	CE/PE	Adebrelimab + apatinib	PFS	Enrolling by invitation
NCT06992609	LS-SCLC	3	70	cCRT or sCRP→	TRT	CE/P	Durvalumab	AE	Recruiting

				IT		E			
NCT07010263	LS-SCLC	3	480	cCRT → IT	TRT	CE/PE	AK112 *vs*. placebo	PFS	Not yet recruiting

Trails in ClinicalTrials. gov were screened by "Small cell lung cancer, limited stage" in the condition section and "Radiotherapy or chemoradiotherapy or radiation" in the intervention/treatment section. The interventional studies with immunotherapy were enrolled in the Supplementary Table 2. The table was lasted update by November 2025.

CE: carboplatin-etoposide, PE: cisplatin-etoposide; AE: adverse event; cCRT: concurrent chemoradiotherapy; CT: Chemotherapy; PBC: platinum-based chemotherapy; TRT: thoracic radiotherapy; ORR: objective response rate; Pts: patients; RT: radiotherapy; IT: immunotherapy; mPFS: median progression-free survival; mOS: median overall survival; NSCLC: non-small cell lung cancer; G: Grade; pne: pneumonitis; RP: radiation pneumonitis; sCRT: sequential chemoradiotherapy; TRAE: treatment-related adverse events
